# Validation of CRISPRoff guide RNAs for stable repression of human AKT1

**DOI:** 10.17912/micropub.biology.002320

**Published:** 2026-09-25

**Authors:** Yudai Takahashi, Hidenori Matsuzaki

**Affiliations:** 1 Graduate School of Comprehensive Scientific Research, Prefectural University of Hiroshima, Hiroshima, 34, Japan; 2 Department of Life and Environmental Sciences, Faculty of Bioresource Sciences, Prefectural University of Hiroshima, Hiroshima, 34, Japan

## Abstract

CRISPRoff enables stable gene repression through targeted DNA methylation, but its efficiency depends on guide RNA selection. To identify effective guide RNAs for human AKT1, we evaluated ten guide RNAs targeting the AKT1 promoter region in HEK293 cells. Guide RNA g1 produced the strongest reduction in Akt1 protein expression, whereas g4, g5, g7, and g9 also reproducibly reduced Akt1 levels. Interestingly, closely spaced or partially overlapping guide RNAs exhibited markedly different silencing efficiencies. These validated guide RNAs provide a useful resource for future studies of Akt signaling.

**Figure 1. Validation of CRISPRoff guide RNAs targeting the human AKT1 promoter:** (A) Schematic representation of the AKT1 5′ regulatory region showing the positions of the ten guide RNAs (g1–g10), the CpG island, FANTOM5 promoters (p1/p2), and exon 1. Target positions are indicated relative to the major FANTOM5 promoter (p1; +1). (B) HEK293 cells were co-transfected with the CRISPRoff expression plasmid and individual guide RNA expression plasmids. Following puromycin selection and expansion, cells were analyzed by immunoblotting using anti-Akt1 and anti-GAPDH antibodies. (C) Quantification of Akt1 protein levels normalized to GAPDH. Values are expressed relative to the GAL4 control, which was set to 100%. Bars represent the mean ± SD of three independent experiments.

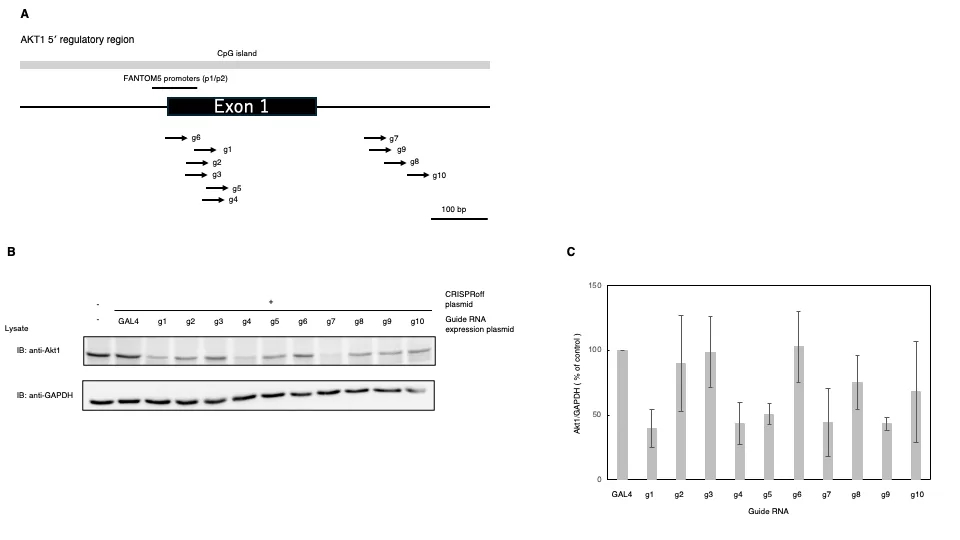



## Description

CRISPRoff is an epigenome-editing system that establishes stable transcriptional repression through targeted DNA methylation without altering the underlying genomic DNA sequence (Nuñez et al., 2021). Because the efficiency of CRISPRoff-mediated repression depends on guide RNA selection, experimentally validated guide RNAs are required for efficient silencing of individual target genes. The Akt family consists of three closely related serine/threonine kinases, AKT1 (PKBα), AKT2 (PKBβ), and AKT3 (PKBγ), which share many functions in regulating cell survival, proliferation, and metabolism while also exhibiting distinct isoform-specific roles (Manning and Toker, 2017). Although several pharmacological Akt inhibitors are available, they generally inhibit multiple Akt isoforms rather than AKT1 specifically (Heerding et al., 2008). In addition, unlike transient siRNA-mediated knockdown, CRISPRoff has the potential to establish stable and long-term gene repression through DNA methylation. Therefore, efficient guide RNAs for CRISPRoff-mediated silencing of AKT1 would provide a useful tool for studies of Akt signaling. However, guide RNAs optimized for CRISPRoff-mediated repression of the human AKT1 gene have not been reported.

The major AKT1 promoter (p1@AKT1) was identified based on the FANTOM5 promoter atlas, in which transcription start sites and promoter activity were mapped using cap analysis of gene expression (CAGE) (FANTOM Consortium and the RIKEN PMI and CLST, 2014). The major FANTOM5 promoter and exon 1 of AKT1 are located within a CpG island, a genomic feature frequently associated with gene promoters (Deaton and Bird, 2011). The CpG island region was defined according to the CpG Islands annotation in the UCSC Genome Browser, which identifies CpG islands based on sequence length, GC content, and the observed-to-expected CpG ratio (Gardiner-Garden and Frommer, 1987). We therefore focused our guide RNA design on the promoter region surrounding the major FANTOM5 promoter. Candidate guide RNAs were identified using the IDT CRISPR-Cas9 guide RNA design tool based on the genomic sequence surrounding the major FANTOM5 promoter. Ten guide RNAs were selected considering their predicted on-target activity, off-target potential, and distribution across the promoter region. Their silencing activities were evaluated by co-transfecting HEK293 cells with CRISPRoff-v2.1 and individual guide RNA expression plasmids, followed by puromycin selection, expansion, and immunoblot analysis of endogenous Akt1 protein expression. Among the ten guide RNAs tested, g1 produced the strongest reduction in Akt1 protein levels. Guide RNAs g4, g5, g7, and g9 also reproducibly reduced Akt1 expression, whereas the remaining guide RNAs showed weaker or more variable effects.

Interestingly, guide RNAs targeting closely spaced or partially overlapping genomic sites exhibited markedly different silencing efficiencies. For example, g1, g2, and g3 target overlapping sequences within the AKT1 promoter, yet g1 produced the strongest repression whereas g2 and g3 showed weaker or more variable effects. Previous studies have shown that CRISPRoff activity is influenced by the position of the guide RNA target site relative to the transcription start site (Nuñez et al., 2021). However, the marked differences observed among closely spaced or partially overlapping guide RNAs in the present study suggest that target position alone does not fully determine CRISPRoff silencing efficiency. Guide RNA activity in CRISPR-Cas9 systems is also known to depend on sequence-related features (Moreno-Mateos et al., 2015), suggesting that sequence-dependent differences in Cas9–guide RNA targeting efficiency may contribute to the variation observed here. Consequently, experimental evaluation of multiple candidate guide RNAs remains important, even when only a limited promoter region is available for guide RNA design.

Together, these results establish a CRISPRoff-based tool for stable repression of human AKT1. The validated guide RNAs reported here provide a useful resource for future studies of Akt signaling and should facilitate the development of CRISPRoff-based experimental systems for investigating isoform-specific functions of AKT1.

## Methods

Cell culture and transfection HEK293 cells were cultured in Dulbecco's modified Eagle's medium (DMEM) supplemented with 10% fetal bovine serum, 100 U/mL penicillin, and 100 μg/mL streptomycin at 37°C in a humidified atmosphere containing 5% CO₂. Cells were co-transfected with CRISPRoff-v2.1 and the indicated guide RNA expression plasmids using polyethylenimine MAX (PEI MAX; Polysciences). Twenty-four hours after transfection, puromycin (4 μg/mL) was added to the culture medium. Cells were selected for 5–7 days until all non-transfected control cells had been eliminated and were then expanded for an additional 1–2 weeks before analysis. Immunoblotting Cells were lysed directly in SDS sample buffer. Protein concentrations were determined using an SDS-compatible Bradford protein assay. Equal amounts (20 μg) of total protein were subjected to SDS-PAGE and immunoblotting using anti-Akt1 (C73H10, #2938; Cell Signaling Technology) and anti-GAPDH (#60004-1-Ig; Proteintech) antibodies. After incubation with HRP-conjugated secondary antibodies, immunoreactive bands were visualized using Chemi-Lumi One Super (Nacalai Tesque, Kyoto, Japan) and detected with a Luminograph III imaging system (ATTO, Tokyo, Japan). Band intensities were quantified using ImageJ (NIH). Quantitative data represent three independent experiments.

## Reagents

Plasmids

CRISPRoff-v2.1 (Addgene #167981) Ten guide RNA expression plasmids (pSG-puro-g1–g10) targeting the human AKT1 promoter were generated in this study. Guide RNA sequences are provided in the table below.

Plasmids and guide RNAs used in this study. The table lists the names, genotypes, and sources of the plasmids used in this study, together with the target gene and spacer sequence (5′→3′) of each guide RNA.

**Table:** 

**Plasmid**	**Genotype**	**Description**
**CRISPRoff-v2.1**	CAG::DNMT3A-DNMT3L-XTEN80-dCas9-HA-2×NLS-BFP-KRAB	Available from Addgene (#167981).
**pSG-puro**	hU6::gRNA; hPGK::EGFP-P2A-Puro::SV40 poly(A)	Guide RNA expression plasmid backbone. Constructed in this study using a custom plasmid synthesized by VectorBuilder.
		
**Plasmid**	Target	Spacer sequence(5’→3’)
**pSG-puro-GAL4**	GAL4	GAACGACTAGTTAGGCGTGTA
**pSG-puro-AKT1-g1**	Human Akt1	GGCCCGAAGACGGGAGCAGG
**pSG-puro-AKT1-g2**	Human Akt1	ACGCAGCGCGGCCCGAAGAC
**pSG-puro-AKT1-g3**	Human Akt1	CACGCAGCGCGGCCCGAAGA
**pSG-puro-AKT1-g4**	Human Akt1	GAGCACCGAGCGCTGGGCAC
**pSG-puro-AKT1-g5**	Human Akt1	AGCACCGAGCGCTGGGCACC
**pSG-puro-AKT1-g6**	Human Akt1	GCAGGACCGAGCGCGGCAG
**pSG-puro-AKT1-g7**	Human Akt1	CCGCGGCGCCGCCAGAATGG
**pSG-puro-AKT1-g8**	Human Akt1	GGAAGTGGCCGAGCGGGCCT
**pSG-puro-AKT1-g9**	Human Akt1	CGCCGCCAGAATGGAGGAGC
**pSG-puro-AKT1-g10**	Human Akt1	CGCGCGGGCCCGGCCAAGGG

Cell line: HEK293 (ATCC CRL-1573)

## References

[R1] Deaton AM, Bird A (2011). CpG islands and the regulation of transcription.. Genes Dev.

[R2] Forrest AR, Kawaji H, Rehli M, Baillie JK, de Hoon MJ, Haberle V, Lassmann T, Kulakovskiy IV, Lizio M, Itoh M, Andersson R, Mungall CJ, Meehan TF, Schmeier S, Bertin N, Jørgensen M, Dimont E, Arner E, Schmidl C, Schaefer U, Medvedeva YA, Plessy C, Vitezic M, Severin J, Semple C, Ishizu Y, Young RS, Francescatto M, Alam I, Albanese D, Altschuler GM, Arakawa T, Archer JA, Arner P, Babina M, Rennie S, Balwierz PJ, Beckhouse AG, Pradhan-Bhatt S, Blake JA, Blumenthal A, Bodega B, Bonetti A, Briggs J, Brombacher F, Burroughs AM, Califano A, Cannistraci CV, Carbajo D, Chen Y, Chierici M, Ciani Y, Clevers HC, Dalla E, Davis CA, Detmar M, Diehl AD, Dohi T, Drabløs F, Edge AS, Edinger M, Ekwall K, Endoh M, Enomoto H, Fagiolini M, Fairbairn L, Fang H, Farach-Carson MC, Faulkner GJ, Favorov AV, Fisher ME, Frith MC, Fujita R, Fukuda S, Furlanello C, Furino M, Furusawa J, Geijtenbeek TB, Gibson AP, Gingeras T, Goldowitz D, Gough J, Guhl S, Guler R, Gustincich S, Ha TJ, Hamaguchi M, Hara M, Harbers M, Harshbarger J, Hasegawa A, Hasegawa Y, Hashimoto T, Herlyn M, Hitchens KJ, Ho Sui SJ, Hofmann OM, Hoof I, Hori F, Huminiecki L, Iida K, Ikawa T, Jankovic BR, Jia H, Joshi A, Jurman G, Kaczkowski B, Kai C, Kaida K, Kaiho A, Kajiyama K, Kanamori-Katayama M, Kasianov AS, Kasukawa T, Katayama S, Kato S, Kawaguchi S, Kawamoto H, Kawamura YI, Kawashima T, Kempfle JS, Kenna TJ, Kere J, Khachigian LM, Kitamura T, Klinken SP, Knox AJ, Kojima M, Kojima S, Kondo N, Koseki H, Koyasu S, Krampitz S, Kubosaki A, Kwon AT, Laros JF, Lee W, Lennartsson A, Li K, Lilje B, Lipovich L, Mackay-Sim A, Manabe R, Mar JC, Marchand B, Mathelier A, Mejhert N, Meynert A, Mizuno Y, de Lima Morais DA, Morikawa H, Morimoto M, Moro K, Motakis E, Motohashi H, Mummery CL, Murata M, Nagao-Sato S, Nakachi Y, Nakahara F, Nakamura T, Nakamura Y, Nakazato K, van Nimwegen E, Ninomiya N, Nishiyori H, Noma S, Noma S, Noazaki T, Ogishima S, Ohkura N, Ohimiya H, Ohno H, Ohshima M, Okada-Hatakeyama M, Okazaki Y, Orlando V, Ovchinnikov DA, Pain A, Passier R, Patrikakis M, Persson H, Piazza S, Prendergast JG, Rackham OJ, Ramilowski JA, Rashid M, Ravasi T, Rizzu P, Roncador M, Roy S, Rye MB, Saijyo E, Sajantila A, Saka A, Sakaguchi S, Sakai M, Sato H, Savvi S, Saxena A, Schneider C, Schultes EA, Schulze-Tanzil GG, Schwegmann A, Sengstag T, Sheng G, Shimoji H, Shimoni Y, Shin JW, Simon C, Sugiyama D, Sugiyama T, Suzuki M, Suzuki N, Swoboda RK, 't Hoen PA, Tagami M, Takahashi N, Takai J, Tanaka H, Tatsukawa H, Tatum Z, Thompson M, Toyodo H, Toyoda T, Valen E, van de Wetering M, van den Berg LM, Verado R, Vijayan D, Vorontsov IE, Wasserman WW, Watanabe S, Wells CA, Winteringham LN, Wolvetang E, Wood EJ, Yamaguchi Y, Yamamoto M, Yoneda M, Yonekura Y, Yoshida S, Zabierowski SE, Zhang PG, Zhao X, Zucchelli S, Summers KM, Suzuki H, Daub CO, Kawai J, Heutink P, Hide W, Freeman TC, Lenhard B, Bajic VB, Taylor MS, Makeev VJ, Sandelin A, Hume DA, Carninci P, Hayashizaki Y, FANTOM Consortium and the RIKEN PMI and CLST (DGT) (2014). A promoter-level mammalian expression atlas.. Nature.

[R3] Gardiner-Garden M, Frommer M (1987). CpG islands in vertebrate genomes.. J Mol Biol.

[R4] Heerding DA, Rhodes N, Leber JD, Clark TJ, Keenan RM, Lafrance LV, Li M, Safonov IG, Takata DT, Venslavsky JW, Yamashita DS, Choudhry AE, Copeland RA, Lai Z, Schaber MD, Tummino PJ, Strum SL, Wood ER, Duckett DR, Eberwein D, Knick VB, Lansing TJ, McConnell RT, Zhang S, Minthorn EA, Concha NO, Warren GL, Kumar R (2008). Identification of 4-(2-(4-amino-1,2,5-oxadiazol-3-yl)-1-ethyl-7-{[(3S)-3-piperidinylmethyl]oxy}-1H-imidazo[4,5-c]pyridin-4-yl)-2-methyl-3-butyn-2-ol (GSK690693), a novel inhibitor of AKT kinase.. J Med Chem.

[R5] Manning BD, Toker A (2017). AKT/PKB Signaling: Navigating the Network.. Cell.

[R6] Moreno-Mateos MA, Vejnar CE, Beaudoin JD, Fernandez JP, Mis EK, Khokha MK, Giraldez AJ (2015). CRISPRscan: designing highly efficient sgRNAs for CRISPR-Cas9 targeting in vivo.. Nat Methods.

[R7] Nuñez JK, Chen J, Pommier GC, Cogan JZ, Replogle JM, Adriaens C, Ramadoss GN, Shi Q, Hung KL, Samelson AJ, Pogson AN, Kim JYS, Chung A, Leonetti MD, Chang HY, Kampmann M, Bernstein BE, Hovestadt V, Gilbert LA, Weissman JS (2021). Genome-wide programmable transcriptional memory by CRISPR-based epigenome editing.. Cell.

